# Your Personal Motivator is with You: A Systematic Review of Mobile Phone Applications Aiming at Increasing Physical Activity

**DOI:** 10.1007/s40279-019-01128-3

**Published:** 2019-05-29

**Authors:** Masoumeh Hosseinpour, Ralf Terlutter

**Affiliations:** Department of Marketing and International Management, Alpen-Adria-Universität, Klagenfurt, Universitätsstrasse 65-67, 9020 Klagenfurt, Austria

## Abstract

**Background:**

Literature shows mixed evidence about the power of mobile phone applications to foster physical activity. A systematic integration that offers insights into which mobile phone application techniques can or cannot foster physical activity is lacking, as is a theoretical integration of current research.

**Objectives:**

We performed a systematic review guided by a theoretical framework focusing on effects that certain mobile phone application techniques have on physical activity, to improve our understanding of what techniques are more or less effective.

**Methods:**

We identified articles by searching EBSCO Business Source Complete, Science Direct, PsycINFO, Springer, PLoS ONE, Taylor and Francis, IEEE, Social Science Citation Index, Science Citation Index Expanded, PUBMED, MEDLINE, and Google Scholar. We considered articles if (1) they referred to the use of mobile phone applications to promote physical activity; (2) their methodological approach allowed one to derive appropriate results (e.g., intervention-based approach, observational study); (3) they were published in peer-reviewed journals or conference proceedings; and (4) they were written in English. The literature search resulted in 41 usable studies. Meta-synthesis and vote counting were applied to analyze these studies.

**Results:**

Based on the ratio of supportive versus non-supportive evidence in both the qualitative and the quantitative studies, we propose the following descending rank order for the effectiveness of application techniques to foster physical activity. This is tentative in nature because the current overall small body of literature made coming to definite conclusions difficult: (1) feedback, (2) goal setting and its sub-forms, (3) competition, social sharing with familiar users in both segregated and social network groups, and (4) social sharing with strangers in segregated groups, reward, and social sharing with strangers in social network groups. Rewards in particular provided mixed results, and social sharing with strangers in segregated and social network groups seemed rather ineffective but may work under special conditions that need to be identified in additional research. One limitation of our study was that our results are mostly derived from qualitative studies, since quantitative studies are underrepresented in the field.

**Conclusion:**

Several mobile phone application techniques were identified that have the potential to foster physical activity, whereas others were identified that are unlikely to increase physical activity. Major avenues for future research include more theoretical development and more quantitative studies, among others.

**Electronic supplementary material:**

The online version of this article (10.1007/s40279-019-01128-3) contains supplementary material, which is available to authorized users.

## Key Points

**Table:** 

Overall, feedback, goal setting (both high and low levels), social sharing with familiar users, in either segregated or social network groups, and competition seem to be the most effective techniques in promoting physical activity.
High perceived ease of use, high perceived usefulness, and positive attitudes toward mobile phone applications strengthen the effects of mobile phone applications’ techniques on physical activity.
The research field is characterized by unelaborated theoretical development and in terms of methodology by many qualitative and few quantitative studies.

## Introduction

Physical activity (PA) improves physical and mental health and reduces disease risks and overall premature mortality [1, 2]. According to recent findings, insufficient PA is one of the leading risk factors globally for adult mortality [3] and diseases such as diabetes, colon cancer or breast cancer [4, 5]. With increasing burdens caused by insufficient PA, there is a need to deliver behavior-change interventions to the public at low cost [6, 7].

To nurture people being active, considerable potential lies in electronic health (eHealth) and mobile health (mHealth) technologies [4]. Vandelanotte et al. [7] categorize eHealth and mHealth as the internet, mobile devices, and smartphone and mobile phone applications (apps) [8]. Among these technologies, smartphone and mobile phone apps are particularly attractive because they are widely adopted, people are strongly attached to them, and they exhibit powerful technical capabilities [9, 10]. Apps also hold the promise of producing behavior change and have been used, for instance, to assist patients with cancer increase their PA [11]. A large number of mobile and smartphone users are interested in health apps in general [12] and apps for PA in particular [13]. Against this background, the opportunity to deliver PA-related behavior-change interventions via apps appears intriguing and is increasingly leveraged [14, 15].

Consequently, researchers have shown strong interest in determining whether apps can promote PA, and a large volume of work has recently emerged. However, it has produced mixed results. Some studies report apps can motivate users, enhance their self-efficacy, and foster PA [16–18], but others have failed to find significant effects [19, 20]. There can be many variables determining the effectiveness of apps; one reason for the mixed results may be the different behavior-change techniques (BCTs) used in these apps. Some offer techniques such as virtual competition, virtual rewards, feedback, and goal setting, whereas others allow users to socially share progress with peers. Other variables potentially explaining deviating results are user related, such as users’ perceptions of the app techniques and their demographic characteristics.

To date, five reviews have been conducted and offer insights on apps and PA [15, 21–24]. Although these are informative, it is necessary to go beyond the present scope of summaries, for three reasons. First, the combined assessment by Bort-Roig et al. [15] and Muntaner et al. [22] suggested that approximately half the individual studies discerned a positive effect of apps on PA or health-related behavior, and half did not. Hence, it is necessary to steer away from the app as a whole and direct attention to specific app techniques and their effectiveness in fostering PA. An indication that techniques are decisive is given in selected findings from the results compilation by Bort-Roig et al. [15]. Second, among the available reviews, only the analysis by Matthews et al. [21] was guided by a theoretical framework. Specifically, the authors use persuasive systems design, which implies a description of the design elements of apps analyzed so far, but they do not relate the app techniques to PA outcomes. Hence, the lack of conceptualization on an aggregate level of a link between app techniques and PA represents another important area in which to expand current reviews. Third, since app technology is developing rapidly as more research is conducted, additional studies have been carried out during recent years that warrant inclusion. Thus, it appears worthwhile to provide an up-to-date review of the current status of the literature.

The present research makes four contributions. First, we review the effects of specific app techniques on motivation, self-efficacy, and PA to get insights about what app techniques are more or less effective. Our findings allow us to explain some of the mixed results and to conclude that apps that feature the “right” techniques appear effective in promoting PA. Second, we propose a conceptual framework, based on the BCT taxonomy [25], for analyzing the effects of app techniques on PA. Our conceptual framework offers insights suggesting that (in-)effectiveness of app techniques may further be explained by user-related variables, particularly by users’ perceptions of apps. Proving feasible for an integration of findings, the framework may also serve pending research as theoretical guidance. Third, we provide a comprehensive tabular description of 41 individual studies identified for our review (Electronic Supplementary Material [ESM] 1). This compilation informs about the results and characteristics of the present research and provides necessary context information to assess the generalizability of the findings. Finally, based on the review, we identify avenues for future research that may guide knowledge development of the field.

## Conceptual Framework

### Overview

**Fig. 1:**
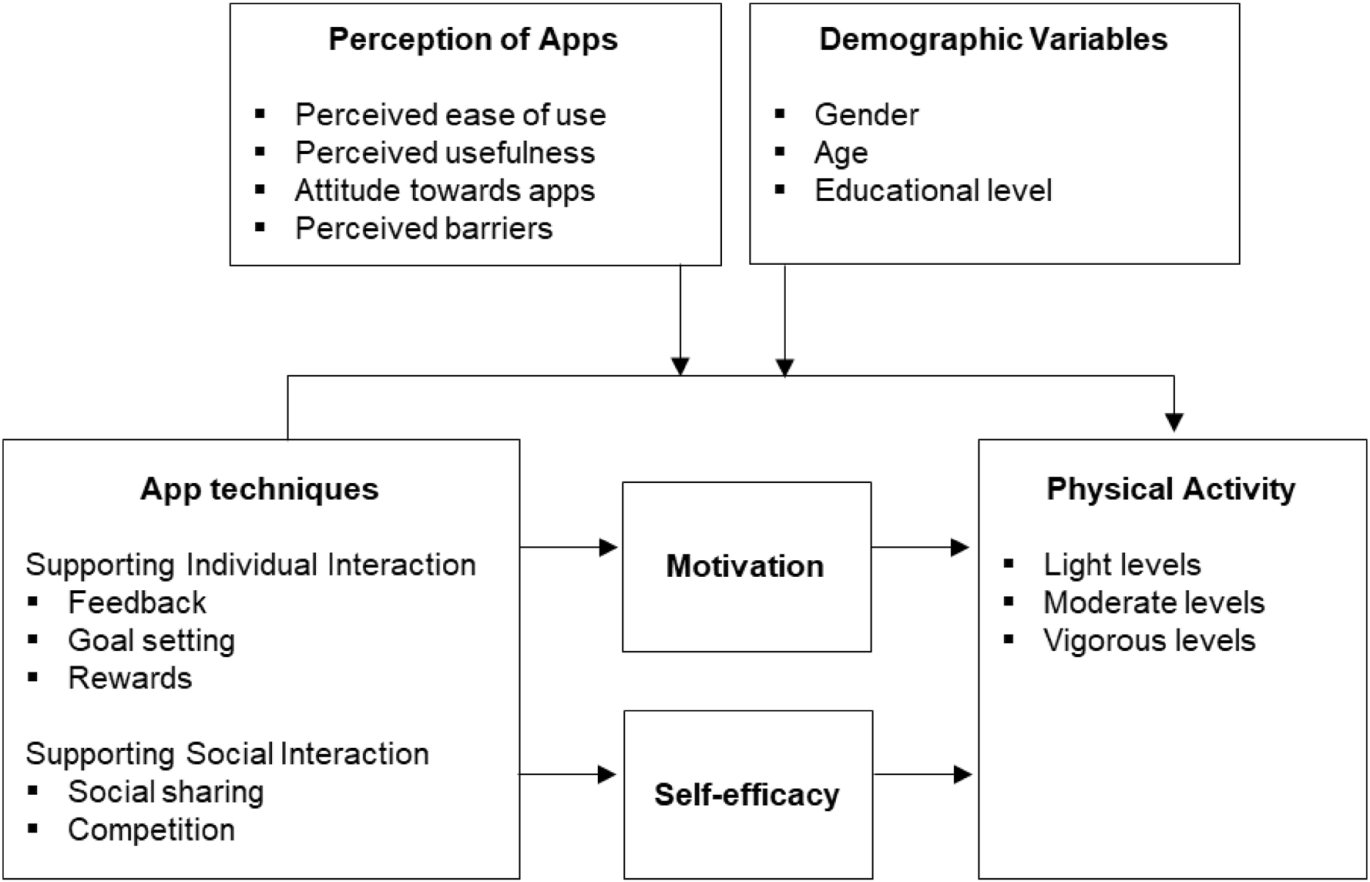
Framework of the effects of app techniques on users’ physical activity, as currently studied in the literature

The conceptual framework for this review is depicted in Fig. 1. Our main objective was to develop a conceptual framework that allowed us to integrate the empirical findings in the research field. Hence, we developed it based on the variables and relationships analyzed in the existing literature. Parts of the framework were also guided by theories in the research field. The app techniques in our framework are based on the BCT taxonomy [25], which synthesizes 19 frameworks of BCTs for describing health-related behavior-change interventions, including interventions to foster PA. The BCT aims to provide a consensually agreed taxonomy of techniques that are used in behavior-change interventions [26, 27]. According to Conroy et al. [28], Hartmann-Boyce et al. [29], Mercer et al. [30], Compernolle et al. [31], and Mansi et al. [32], the most common techniques in the BCT taxonomy are feedback, goal setting, rewards, social sharing, and competition, which we focus on in our review. In addition to these app techniques, our framework comprises mediator and moderator variables. Mediator variables are motivation and self-efficacy. Moderator variables comprise variables capturing users’ perception of the apps (i.e., perceived ease of use, perceived usefulness, attitude toward apps, and perceived barriers) and demographic variables (sex, age, and educational level). PA is the target behavior.

### Current Theories in the Field

To date, theoretical development in the research field is not well-elaborated. The most-often applied theories and concepts are control theory [33], social cognitive theory [34], variables related to technology-acceptance models (but typically not established technology-acceptance models themselves) [35], and self-determination theory [36].

Control theory offers a theoretical basis for understanding health-related behavior, especially PA [37]. This theory posits that behavior is goal driven and that individuals change their behaviors with regard to feedback about the deviation between their actual behaviors and the set goals (or set standards). In case of a gap between individuals’ current performance and the goal, negative feedback functions to reduce or remove the inconsistency, leading people to try harder if they are far from their goals [38]. With regard to fostering PA through apps, the realization of such discrepancies can be stimulated by techniques that facilitate the individual’s reflection on their performance, comprising feedback, goal setting, and reward [39].

Social cognitive theory has been shown to be valuable for explaining health-related behaviors such as PA [40]. It proposes that observational learning can change individuals’ behavior [34]. Observational learning means that individuals learn how to accomplish a specific behavior by creating modeled behavior from the observation of others’ success, which then serves as a guidance and an inspiration for their own task accomplishment [41]. With regard to fostering PA through apps, techniques that allow for supporting social interaction (i.e., social sharing and competition) foster such learning processes [42, 43]. By socially interacting, individuals receive information about the behavior and performance of others, leading them to observe, construct role models, and eventually learn how to successfully perform PA themselves.

According to social cognitive theory, individuals need to understand the potential outcome of a modeled behavior and are more likely to exhibit a behavior if it results in valued outcomes, for instance, rewards [44]. Rewards can lead to feelings such as pride or joy, which enhance the probability of behaving similarly in future to experience those feelings again [45].

Within social cognitive theory, self-efficacy—the individual’s belief in their being able to achieve a goal—is a focal determinant of a given behavior [40, 45]. Self-efficacy can be changed based on efficacy expectations [32, 46] developed based on learning mechanisms, either through personal experience or observations [47]. App techniques can aim at both. If app techniques help individuals master a difficult or feared task, users learn about their capabilities by personal experience, thereby enjoying an increase in perceived self-efficacy. If app techniques illustrate others’ success, they help users learn by observation, potentially raising their aspirations and belief in their own capabilities.

Current literature also analyzes variables that can be attributed to the research stream on technology acceptance [48] and that are valuable in different contexts of new technology adoption, such as collaborative technologies or health information systems [49]. Research mainly discusses two factors: perceived ease of use and perceived usefulness. According to both dimensions, users may perceive app techniques as easy or difficult to work with and as useful or useless in meeting the intended purpose. Depending on these perceptions, users may either accept or reject app usage aiming to foster PA.

Self-determination theory is a theory of human motivation that has been applied in the context of PA behavior [50]. Self-determination theory assumes motivation can be intrinsic or extrinsic. Intrinsic motivation arises from within individuals and pertains to engaging in an activity for its inherent pleasure and satisfaction. Extrinsic motivation is driven by outside stimuli and refers to behavior performed because individuals seek external incentives like fame, praise, or money [36]. Apps can aim at triggering both intrinsic and extrinsic motivations. If app techniques engage users in performing PA due to feelings of pleasantness and satisfaction inherent in the activity, users are primarily intrinsically motivated. If app techniques engage users in performing PA to receive recognition or approval from others (or the app itself), they are primarily extrinsically motivated.

### App Techniques

Based on prior literature and the BCT taxonomy [28–32, 51, 52], we suggest that, to promote PA, app techniques follow two basic approaches, either supporting individuals’ interactions with the app or supporting social interactions with peers. Techniques aimed at supporting individuals’ interactions with the app provide features users can use on their own, comprising feedback, goal setting, and reward. Techniques aimed at supporting social interactions provide users with connections to other users and comprise social sharing and competition.

#### Supporting Individual Interaction

Feedback as an app technique provides users with information about the progress of their actual PA, such as the number of steps taken in a day. Drawing one’s attention to existing information about current behavior can lead to significant behavior changes [53]. For instance, when a user realizes their current PA performance is insufficient, they may aim to detect what needs to be done [54]. Feedback may also show users their real capability to perform PA. Thus, it helps users become motivated, increase self-efficacy, and reduce discrepancies, resulting in performing PA [55].

Goal setting is an action plan to achieve desired results [25]. Goals are indicative, giving direction about what needs to be done and the required efforts. Goal setting increases consciousness of performance and reduces uncertainty [56]. Achieving goals also holds great potential for self-satisfaction, routing the continuance of current behavior [55]. By providing such sensations, having goals as an app technique may motivate users, increase self-efficacy, and facilitate PA behavior.

Rewards as an app technique are instances of positive reinforcement given in return for the performed behavior. Rewards raise awareness about omitted and lost opportunities where the individual could have done something better [57]. They may positively influence motivation, self-efficacy, and PA in several ways. First, rewarded activities are more likely to be re-performed [58]. Second, while punishing users for insufficient PA may hurt their feelings [59], providing reinforcements about performed PA provides joyfulness [51], which can result in enhanced competence and performance [60]. Third, rewards can help users better judge their own abilities. Hence, rewards as an app technique may foster users’ motivation, self-efficacy, and PA performance.

#### Supporting Social Interaction

Social sharing is an app technique allowing users to share their PA performance, such as the number of steps taken, with others in a group, for instance, with friends [61]. Sharing information helps users observe others’ PA performance and create modeled behaviors (while the user’s PA performance is simultaneously visible to others). Social sharing may aim to increase motivation and self-efficacy, since observing others’ behaviors helps individuals become inspired and evaluate their own ability to perform (more) PA. Thus, social sharing can provide access to learning about others’ behavior and how to accomplish their own tasks, resulting in heightened levels of motivation, self-efficacy, and PA [62–64].

Competition as an app technique refers to the process of evaluating one’s own abilities by comparing them to those of others [65, 66]. It is a mechanism for increasing individuals’ receptiveness to positive behavioral influences in a social context [67]. In this context, if users do not perform sufficient activities, they may be inspired to be more active and not lose the competition [18]. When performing sufficient PA, the good feeling of winning may inspire users to continue or even perform more activities. Competition can also help users assess their own capabilities. Hence, it can motivate them, increase their self-efficacy, and improve levels of PA [68, 69].

### Perceptions of Apps, Motivation, and Self-Efficacy

Perceptions of apps comprise perceived ease of use, perceived usefulness, attitude toward apps, and perceived barriers. These variables may moderate the effects of the app techniques on PA [70–74]. Motivation and self-efficacy are regarded as mediator variables between app techniques and PA.

#### Perception of Apps

Perceived ease of use refers to the degree to which a person believes using a new technology would be effortless [35]. Users have more interest in easy-to-use apps and techniques that can be learned effortlessly [75]. Perceived usefulness refers to the degree to which a person believes using a new technology would enhance their performance [76]. An app technique’s effect is increased if its functions are perceived as useful, for instance, if it allows reliable measurement of PA [77]. So higher perceived ease of use and perceived usefulness expectancy is likely to strengthen the effects of app techniques on PA.

Attitude toward the app reflects people’s favorable or unfavorable beliefs about the app and is primarily an affective evaluation [78]. Attitude is also considered by technology-acceptance models; more favorable attitudes toward the app typically lead to stronger effects of the app techniques on PA [79].

Perceived barriers can impede acceptance of new technologies generally [80] and of apps particularly [81, 82]. Usage barriers occur when an innovation is incongruent with existing practices or habits [83]. For many people, learning to use and cope with the technology renders the perceived likelihood of failure greater than the likelihood of success [84]. Thus, lower perceived barriers strengthen the effects of app techniques on PA.

#### Motivation

Motivation is a psychological process giving behavior purpose and direction [85]. Without motivation, users are unlikely to perform PA [86]. Previous PA studies demonstrated positive associations between motivation and PA behavior [87], and evidence exists that motivation may play a mediating role between app techniques and PA [88–90]. Motivation is a key element when designing apps [54]. In particular, Ryan et al. [91] suggested that individuals’ motivation to perform PA relates to appearance, health, fitness, challenge, enjoyment, and competence. App techniques may address each of these motivations. For instance, when users decide to use the app to perform (more) PA, the motivational factors involved may relate to appearance, health, and fitness. Social and individual functionality provided by app techniques may also speak to the motivational factors of challenge, enjoyment, and competence.

#### Self-Efficacy

Self-efficacy relates to individuals’ beliefs and judgments about their capabilities to perform a specific behavior in a given situation [34]; previous studies have demonstrated positive associations between self-efficacy and PA behavior [92]. Further, self-efficacy may play a mediating role between the app techniques and PA [88, 93, 94]. If individuals have the impression that they have the ability to perform PA (i.e., high perceived self-efficacy), they are more likely to actually engage in PA behavior. App techniques may convey such impressions. For instance, through positive feedback from the app, individuals may realize their real ability to perform PA or get a feeling of gaining control over situations formerly preventing them from performing PA, such as lack of time and/or routines in daily life.

### Physical Activity

PA can be defined as “athletic, recreational or occupational activities that require physical skills and utilize strength, power, endurance, speed, flexibility, and range of motion or agility” [95]. PA can be distinguished on light, moderate, and vigorous levels. Light activities are simple activities such as housework, travel behavior (i.e., frequency of public transport usage), taking stairs, and taking a break from sitting [17, 96]. Moderate activities are more intensive and include walking, taking more steps, and physically demanding household activities such as chopping wood [18, 94, 97]. Vigorous activities reflect sport activities with high intensity levels, such as swimming, cycling, jogging, running, and fitness activities [19, 98].

### Demographic Variables

We consider sex, age, and educational level as moderating variables, since they are behaviorally relevant regarding technology. For instance, on one hand, males tend to have lower levels of anxiety with technology than females and are more eager to accept new technological devices [99, 100]. On the other hand, women tend to be more health conscious than men [101]. Younger people favor online shopping more than do older people [102], and better-educated people tend to have greater ability to learn in new environments than less-educated people and find it easier to use the internet [103].

## Methods

### Literature Search

Following the guidelines by Roschk et al. [104] and Liberati et al. [105] for systematic reviews, we conducted a a computerized bibliographic search using the keywords physical activity, exercise, mobile phone application, smartphone applications, self-efficacy, and motivation. Searched databases included EBSCO Business Source Complete, Science Direct, PsycINFO, Springer, PloS ONE, Taylor and Francis, IEEE, Social Science Citation Index, Science Citation Index Expanded, PUBMED, MEDLINE, and Google Scholar [106]. In addition, we (1) consulted the references of previous reviews; (2) searched the Social Science Citation Index, the Science Citation Index Expanded, and Google Scholar for articles referring to these previous reviews; and (3) scanned the reference lists of articles we found to identify potential additional articles.

### Selection Criteria

To be included in the review, articles were required to meet the following criteria: (1) they referred to using apps to promote PA; (2) their methodological approach allowed us to derive results about the effects of app techniques on PA (e.g., intervention-based approach, observational study); (3) they were published in peer-reviewed journals or conference proceedings; and (4) they were written in English. We did not consider studies if (1) the full text was not available or (2) the study was irrelevant. The two authors independently conducted article selection, and disagreements were resolved through discussion.

### Data Analysis Methods

To provide a comprehensive understanding of studies in this field, the present systematic review aims to summarize both qualitative and quantitative data, for which different approaches exist. We applied two approaches, meta-synthesis and vote counting, following recommendations by Cooper [107], Zimmer [108], and Bushman and Wang [109].

Meta-synthesis is a methodological technique aiming to provide an overview of existing studies at a more abstract level than individual studies, based on processes of synthetization and interpretative translation of findings [108]. In synthetization, all selected studies are thoroughly reviewed, key findings are extracted, and an integrative account reflecting the findings’ similarities and differences is developed. Interpretative translation parallels this process, aiming to understand findings and their (dis)similarities through an interpretative lens against the studies’ individual and heterogeneous backgrounds (e.g., assumptions, methodological approaches) [110]. Meta-synthesis is typically applied to interpret findings in qualitative research, yet it can also be applied to quantitative research (see for example, Byun et al. [111] and Tokunaga [112]).

Vote counting is a methodological technique of categorizing and analyzing conflicting results by comparing the frequency of supportive findings against that of non-supportive findings [107, 109, 113]. Traditionally, vote counting is applied in quantitative studies but can also be applied to evaluate qualitative data (see Leamy et al. [114]). Even when primary data provide insufficient information to calculate effect sizes for meta-analysis (typically the case with qualitative data), these data and results may still provide sufficient information for a frequency-based account of supportive versus non-supportive findings.

### Data Extraction

In the appendix (ESM 1), we provide a tabular description of the 41 individual studies identified for our review. For the selected articles, we extracted details on source (author names, publication year, and country of the first author’s university), type of PA, target group, sample size, and demographic characteristics (i.e., sample’s average age, sex composition, and educational level). We also extracted data on studies’ theoretical approaches, methodological approaches (research design, nature of results in qualitative or quantitative terms), data-collection approaches, analysis approaches, timeframe, and app techniques used to promote PA. We also compiled the attitudinal and psychological variables the studies referred to.

To complete our database, we screened studies’ results and extracted information in terms of the effects of app techniques on users’ motivation, self-efficacy, and PA behavior. We also screened results for findings on mediation effects of motivation and self-efficacy between app techniques and PA. Finally, we gathered results about moderating effects of users’ perceptions of app techniques, sex, age, and educational level.

## Results

### Study Selection

**Fig. 2:**
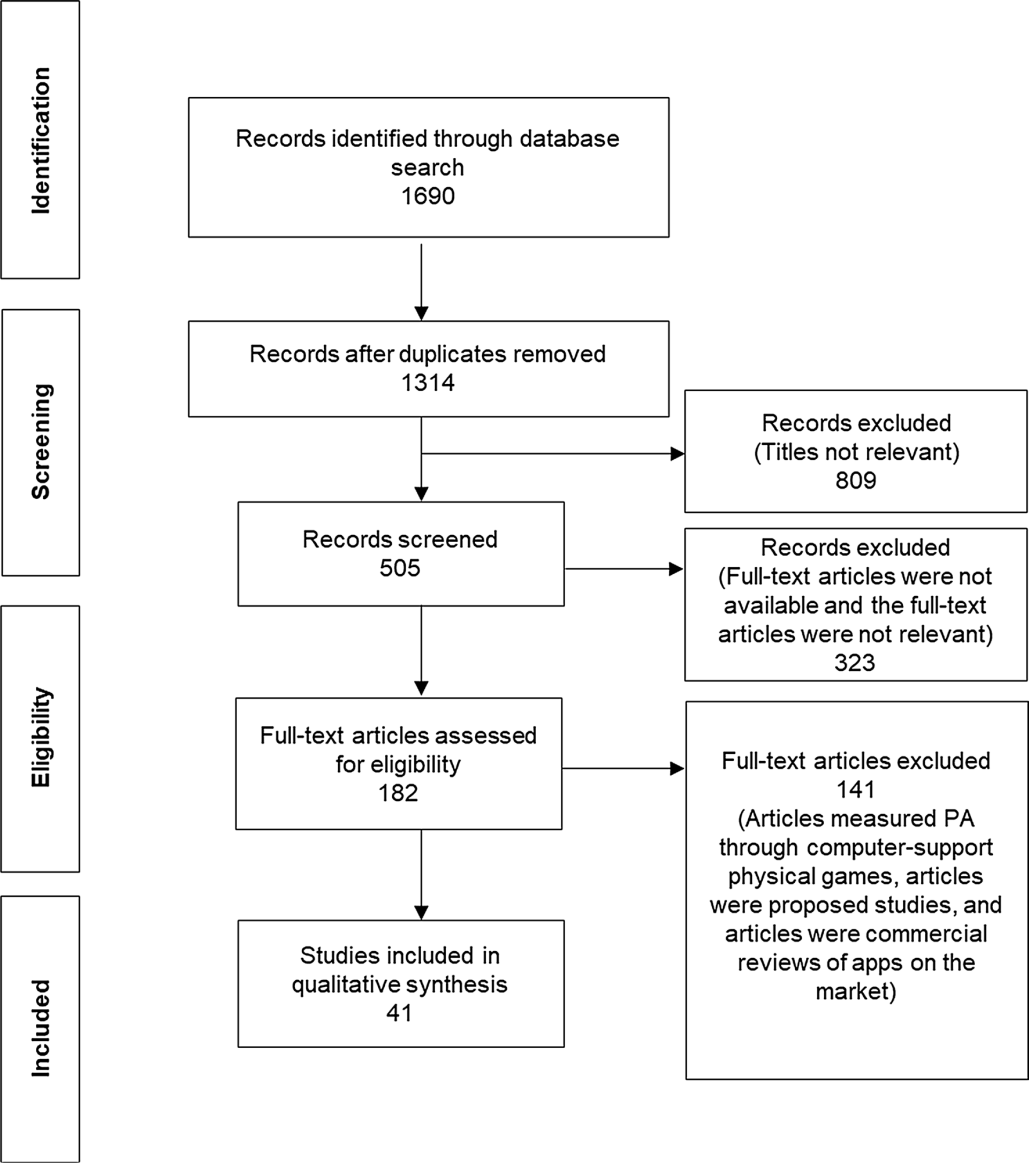
Search and exclusion process. App techniques and physical activity (PA)

The literature search covered 2002–2018 and yielded 1690 abstracts. First, we removed duplicates. Second, articles with irrelevant titles were removed, leaving 505 unique, potentially relevant abstracts (Fig. 2). Third, after excluding records outside the scope (i.e., falling under the exclusion criteria), the full texts of 182 records were checked. Of these, 141 did not meet the inclusion criteria. The main reasons were (1) they focused on promoting PA through computer-supported physical games; (2) they were proposals for studies yet to be conducted; or (3) they were commercial reviews of apps on the market. The literature search resulted in 41 usable articles.

### Study Characteristics

Regarding the sample characteristics, the 41 included studies referred to a combined total of 3553 respondents. Table 1 depicts the studies’ sample characteristics. Male respondents (57.1%) were more represented. Subjects came from different educational backgrounds, with 52.8% mentioning high school as their highest qualification. Ages ranged from 13 to 81 years; the lower average boundary was 26.1 years, and the upper was 50.3 years. In total, 51.2% of the studies analyzed apps targeting a general population, and 48.8% focused on special target groups (e.g., diabetic, obese, or sedentary individuals). About half of the studies had a study duration of < 30 days. All but two studies [54, 115] came from a variety of high-income nations. Studies from Europe, North America, and Australia were well represented (95.2%); Asia (2.4%) and Latin America (2.4%) were underrepresented, and Africa seemed to be missing entirely. However, we concluded this from the first author’s university affiliation, since the geographical origin of the data was rarely provided. Sample sizes were typically rather small; only 29.4% of the studies had > 70 subjects.

**Table 1:** User demographic characteristics across the study samples

Items	Statistics
Average sex share of studies (%)	
Female	42.9
Male	57.1
Target groups of studies (%)	
General population	51.2
Special respondent groups (i.e., diabetics, the obese, joggers, runners, outpatients, veterans, nurses, females, males, and sedentary individuals)	48.8
Sample size of studies (%)	
6–69	70.6
70–133	9.8
134–170	4.9
171–234	4.9
≥ 235	9.8
University’s country of origin (%)	
Europe	46.4
North America	39.0
Australia	9.8
Latin America	2.4
Asia	2.4
Africa	–
Average boundaries of age range (years)	
Lower (min–max)	26.1 (13–52)
Upper (min–max)	50.3 (27–81)
Length of study duration	
≤ 30 days	47.4
≥ 31 days	52.6
Average education level of studies	
High school	52.8
Vocational	8.2
College and bachelor	34.7
Master or doctoral	4.3

Regarding the research paradigm of the 41 studies, 48.8% (*n* = 20) followed qualitative assessments (i.e., verbal, written, or statistical descriptive account carrying information about how the app techniques affected the dependent variables), 14.7% (*n* = 6) embarked on a quantitative approach (e.g., measured mean differences in levels of dependent variables as a function of the app techniques), and 36.5% (*n* = 15) applied a mixed-methods approach combining qualitative and quantitative assessments.

### App Techniques

Across studies, 90.2% (*n* = 37/41) analyzed techniques supporting individuals’ interaction with apps, and 53.7% (*n* = 22/41) analyzed techniques supporting social interactions with peers. The sum of these numbers exceeds 100%, since studies usually used more than one technique in the app design.

#### Feedback

Of the studies, 78.0% (*n *= 32/41) analyzed feedback, considered in a multitude of forms, with some studies using more than one form. Feedback was represented as bar graphs and a virtual map [98, 116–120]; audio feedback from virtual trainers [77]; illustrations through avatars [17, 55, 59, 75, 121, 123–125, 127]; tailored text and email messages [94, 128–130]; real time, self-monitoring, receiving reminders, GPS tracking, tempo of music, and biofeedback [18, 19, 54, 77, 86, 89, 98, 119, 120, 122, 123, 126, 128, 131–135].

Though feedback was illustrated quite differently across studies, most results indicated that feedback positively affected users’ PA. The study by Lin et al. [59] is a prototypical example: feedback is represented through an avatar in the form of a fish. Based on the user’s performance (daily steps), the fish grows and multiplies or turns from “happy” (sufficient progress) to “angry” (nearly sufficient progress) or even “sad” (insufficient progress). In this study, average steps taken was measured using a pedometer. Results indicate that daily steps increased by 20% for 12 of 19 users during the examination period. While studies used one or another form of feedback and PA and differed in methodological designs, using either more (e.g., descriptive pre–post comparison) or less systematic approaches (e.g., self-monitored changes in PA levels, interview results), the results generally indicated that feedback increased PA [18, 126]. These qualitative findings were complemented by quantitative results that found a significant increase in PA in groups that received feedback compared with those that did not [17, 55, 86, 89, 120, 122, 123, 129].

Despite these mostly positive effects, some qualitative studies also indicated there can be negative or null effects. For a small number of users in Lin et al. [59], feedback decreased PA because of emotional connections to the avatar (fish), which backfired because users stopped checking when they saw their fish was sad. Feedback in Duncan et al. [19] and Garcia-Ortiz et al. [134] was non-significant, but unfortunately, no explanation for the absence of effects was provided.

The qualitative findings further indicate that feedback motivates users, because visualizing PA keeps activities in front of their minds and reminds them if discrepancies are present in their goals [75, 119]. Feedback may also increase self-efficacy, as reflected in users’ realizing their capabilities, for instance, expressed by one user in Harries et al. [55], “Actually I walked two miles the other day and it seemed like nothing; I can walk that.” Only in van der Weegen et al. [120] did feedback not foster PA, but no explanation for this was given. Finally, feedback might also create awareness, such as an understanding how inactive or active users actually are and that simple activities like “dog walking” count [75, 121].

From a vote counting perspective, of 14 quantitative studies, 12 versus 2 [19, 134] studies showed significant, positive effects of feedback on PA [17, 55, 77, 86, 89, 120, 122, 123, 127, 129, 133, 135]. The remaining 18 studies reported qualitative, supporting results. Hence, the combined evidence of meta-synthesis and vote counting provides a strong indication that feedback fosters PA. There are also indications that feedback fosters motivation and self-efficacy.

#### Goal Setting

In total, 36.5% (*n* = 15/41) of studies analyzed goal setting. Goal setting is primarily distinguished based on who sets the goal and how challenging the goal level is. Goals can be (1) app-set, based on users’ PA baseline; (2) user-set, with goals set either independently by users or by following researchers’ recommendations; or (3) a mix of app- and user-set, where users choose among app-provided predefined goals. Levels of goal challenges are either higher or lower: (1) higher levels mean more challenging goals are formulated, typically in absolute numbers (e.g., taking 10,000 steps/day), or as challenging increases in PA (e.g., 20% weekly increase in steps/day), or as workout sessions planned by a (virtual) trainer (e.g., running activities, 1 km/session) [16, 18, 56, 94, 98, 119, 128]; (2) lower goals are less challenging and are also formulated as an absolute increase in PA (e.g., 10% weekly increase in steps/day) or as a gradual increase [115, 117, 118, 122, 123, 126, 136, 137].

Distinguishing between app-set, user-set, and mixed-set goals did not allow us to discern meaningful differences in findings, but distinguishing between goal challenge levels proved more substantial. For users with “high” goals, most findings indicated a positive effect on PA. For instance, Boratto et al. [16] found a significant increase in PA in a randomized controlled group setting, and Fukuoka et al. [94] provided descriptive interview results supporting these findings. Despite increased PA, high goals were often perceived by users as too tough and challenging, which might impede positive effects [18, 56, 98, 119]. For instance, a user in Munson and Consolvo [98] claimed feeling “failure” upon not reaching difficult goals, even though they performed PA. In Consolvo et al. [18], users wished to set goals at a lower level, even though they performed more PA with the challenging goals. Collectively, users mostly considered high goals as too ambitious, which could be the reason for the non-significant effects in Fanning et al. [128]. For users receiving “low” goals, studies mostly reported positive effects on PA [115, 122, 123, 137]. Positive effects were also supported by qualitative studies, where users in interviews or focus groups mentioned that they found these goals convincing and doable [118, 126, 136]. Taken together, there are indications that both low and high goal challenge levels increase PA but that goals should not be too difficult or challenging, at least according to user preferences.

The qualitative results also indicated that goal setting motivates users, since it challenges them and provides structured plans acting as “personal trainers” [117].

Based on vote counting, for high goals there were two quantitative studies, one of which provided non-significant effects [128] and another provided supportive results [16]. Four other qualitative studies (of a total of five qualitative studies) [18, 56, 94, 119] provided supportive results, and another qualitative study [98] provided mixed results on PA. For low goals, there were four qualitative and four quantitative studies. All four quantitative studies [115, 122, 123, 137] and four qualitative studies [117, 118, 126, 136] provided supportive effects on PA. Hence, the combined evidence of meta-synthesis and vote counting indicated that goal setting seems to foster PA and that users prefer low over high goals. There are also indications that goal setting fosters motivation. Self-efficacy related to goal setting was not analyzed.

#### Reward

A total of 17.0% (*n* = 7/41) of the studies included rewards such as badges, butterflies, trophies, ribbons, stars ‘*’, electronic postcards [17, 18, 98, 116, 128], encouraging messages, and collectable points [94, 115]. Administration of rewards followed the same pattern in all studies; users received the first reward upon initial PA performance and then further rewards by additional PA performance and attaining goals.

The evidence was marked by mixed results. Fukuoka et al. [94] and Fanning et al. [128] provided supportive evidence for a positive effect of reward on PA. In Fukuoka et al. [94], users received an encouraging message (i.e., “excellent job”) upon PA performance, which they generally evaluated very positively. One user in the interview session said, “I am getting credit for all of it.” These positive results were also supported by a quantitative study [128], which found a statistically significant increase in daily steps from 34.88 to 46.77 min in the intervention group as compared with a control group that received no reward.

However, several studies found evidence that reward does not necessarily support PA. In Ahtinen et al. [116], 60.5% of users reported reward to be ineffective. Further, for 25 of 28 users in Munson and Consolvo [98], reward did not foster PA; for instance, one user claimed, “I really wanted the trophy thing” but that failing to get one was “disappointing”. Likewise, other users said, “not that motivating,” “gimmick,” “lame,” or “stopped caring.” Zuckerman and Gal-Oz [115] did not find significantly higher PA in the reward group compared with a non-reward group.

In studies where reward showed positive effects, it fostered motivation by increasing excitement and encouragement. A user in Consolvo et al. [18] said, “It was like, yes, I rock! And it was fun to go back and go, yes, there’s my star for that day.” Reward also increased self-efficacy, as one user stated, “I could see the butterfly and think, I did it last week, you can do it again this time” [17]. In Fanning et al. [128], the reward group perceived higher self-efficacy than groups without reward.

According to vote counting, three qualitative studies (of a total five qualitative studies) [17, 18, 94] and one quantitative study [128] (of two quantitative studies) showed significant, positive effects of reward on PA. Non-supporting results emerged from the other two qualitative studies [98, 116] and the other quantitative study [115]. Results from the meta-synthesis and vote counting both provide indications that reward in the apps can, but do not necessarily need to, foster PA. Results indicate that the type of reward provided by the app is critical. In cases where rewards had positive effects, it was also positively related to motivation and self-efficacy.

#### Social Sharing

Of the studies, 46.3% (*n* = 19/41) analyzed social sharing, which can occur in segregated (smaller, more intimate) groups or social network groups. Within both segregated and social networks, users could share their PA performance either with familiar users (family, friends, or colleagues) or with strangers. Hence, possible sharing constellations are (1) with familiar users in segregated groups [18, 75, 116, 121, 130]; (2) with familiar users in larger social networks [126, 138–140]; (3) with strangers in segregated groups [55, 56, 59, 119, 122, 123, 125, 127]; and (4) with strangers in larger networks [96, 98].

In Consolvo et al. [18], users sharing their PA with familiar people in segregated groups performed significantly more PA than users in non-sharing groups. These users claimed that sharing made them go for walks with friends instead of previously “just sitting around.” Other studies using different designs with either more (e.g., descriptive pre-post comparison) or less systematic approaches (e.g., interview results) supported these findings [75, 121]. Interestingly, for some users in Anderson et al. [75] and Maitland et al. [121], sharing turned to competition and led them to perform more PA, some even “teasing” each other upon performing more PA. The same pattern was observed for users sharing their PA performance with familiar individuals in larger social networks [126, 138–140].

Sharing with strangers in segregated groups appeared to have mixed effects on PA. Though sometimes considered “awkward,” with users asking themselves, for instance, “why anyone would be interested in their workout” [119], some studies found that sharing with strangers fostered PA [122, 123, 125]. However, others reported insignificant results [55, 127] and mixed effects [56, 59]. Sharing with strangers in social networks was ineffective, with users clearly indicating they were not fond of sharing their PA on Facebook (with strangers) [96, 98].

The qualitative accounts indicated that social sharing can increase users’ motivation through receiving social support and feelings of belonging in the group. These feelings were reported with familiar users in both segregated and social network groups but not with strangers [126, 139]. Social sharing with strangers across segregated and social network groups was “horrendous” and “too broad,” and users “fear appearing boring or boastful” [96, 98]. Regarding sharing in social networks with familiars or strangers, users sometimes also felt disappointment when they did not receive reactions from others (e.g., no likes for a post on Facebook [98]). Social sharing for all groups may also have demotivating effects if other users do not participate, with one user [138] reporting that “other group members’ lack of participation impacted her motivation to take steps”. Effects of social sharing as related to self-efficacy were not examined.

From a vote counting perspective, sharing with familiar users in segregated groups fostered PA in one (of one) quantitative [18] and four (of four) qualitative studies [75, 116, 121, 130]. Sharing with familiar users in social networks fostered PA in one (of one) quantitative study [140] and two (of three) qualitative studies [126, 139], and one reported mixed results [138]. Four qualitative and four quantitative studies investigated sharing with strangers in segregated groups. Social sharing increased PA in two quantitative studies [122, 123] and in one qualitative study [125] and failed to do so in two quantitative studies [55, 127] and one qualitative study [119]. It produced mixed results in two qualitative studies [56, 59]. Hence, results were mixed. In the two available qualitative studies that included sharing with strangers in social networks [96, 98], social sharing was ineffective for PA; no quantitative study has yet addressed this issue. Hence, meta-synthesis and vote counting suggest that social sharing with friends, family, and colleagues in both segregated and social networks seems to foster PA, whereas sharing with strangers produces mixed results in segregated groups and seems ineffective in larger social networks. Motivation also increased while sharing with familiar users in both segregated and social network groups. Insufficient evidence was available for self-efficacy.

#### Competition

In total, 29.2% (*n* = 12/41) of the studies used either group or individual competition [54, 59, 75, 89, 115, 116, 121–123]. While these apps did not connect to an external audience, three offered so-called virtual footrace competitions [126, 139, 140], where users could individually compete with others via Facebook.

Across studies, quantitative results show that competition groups yielded significantly higher PA than comparison groups without competition [89, 122, 123]. These findings are complemented by qualitative studies. While studies used different forms of competition and different methodological designs, most reported results indicating that competition increased PA [54, 75, 121]. One user [75] even asked if she could take the neighbor’s “dog for a walk” just to win the competition.

Despite these positive effects, for some users [115], competition did not significantly improve PA. They reported no interest in comparing themselves against others. In one study [59], even though competition had positive effects for a few users, most deemed it unnecessary. One user stated, “There is enough competition in real life; I don’t really need more.”

The qualitative and quantitative findings indicated that competition motivates because it is “fun” and “enjoyable” for users to compete against each other [75, 116, 121, 126, 139, 140]. One study [89] also explicitly looked at motivation and self-efficacy, with the results indicating that competition significantly increased motivation but failed to increase self-efficacy.

Vote counting reveals seven (of eight) qualitative [54, 75, 116, 121, 126, 139, 140] and three (of four) quantitative studies [89, 122, 123] showing significant, positive effects of competition on PA. One quantitative study [115] produced non-significant results, and one qualitative study [59] provide mixed results. Evidence from meta-synthesis and vote counting indicated that competition is likely to foster PA as well as motivation. Findings for self-efficacy are not yet sufficient to provide valid conclusions.

### Perception of Apps, Motivation, and Self-Efficacy

#### Perceived Ease of Use

In total, 17.0% (*n* = 7/41) of studies considered perceived ease of use [75, 98, 117, 119, 121, 128, 132].

All these studies were qualitative in nature and indicated a moderating effect of perceived ease of use on the relationship between app techniques and PA, despite differing study designs (e.g., interview sessions or focus groups). Results indicate that when perceived ease of use was “high” (vs. “low”), the relationship between app techniques and PA seems stronger (weaker). For instance, for users in one study [121], feedback (in an avatar form) fostered PA, since it was perceived as easy to use. Users in another study [117] also performed more PA, since goal setting was easy for them. One stated, “MC (the app) plans everything and the user does not need to think of the duration or intensity. The application does it for you. You just need to arrange time to do the things that it suggests.”

Following a vote counting approach, all seven qualitative studies provided supportive evidence for a moderating effect of perceived ease of use on the relationship between app techniques and PA. Hence, both meta-synthesis and vote counting evidence indicates perceived ease of use has a moderating effect, as higher ease of use seems to strengthen the impact of the app techniques on PA.

#### Perceived Usefulness

A total of 34.1% (*n* = 14/41) of studies analyzed perceived usefulness as another dimension to users’ perception of the app and its techniques, considered in the form of users’ perception of the app as reliable, accurate, able to help in performing PA, and generally positive [17, 59, 75, 94, 98, 115, 117, 119, 121, 123, 130, 132, 138, 139].

Similar to perceived ease of use, results indicated a moderating role of perceived usefulness on the relationship between app techniques and PA. All studies were qualitative and, despite differing designs (e.g., interview sessions or focus groups), they showed that higher perceived usefulness leads to a stronger relationship between app techniques and PA. Users in one study [75] perceived the app overall to be both reliable and stable when measuring PA, strengthening the effects of the app techniques on PA. Other users perceived the app as useful in making them aware of PA, providing structured plans, and reminding them to engage in PA [17, 75, 121, 138], perceptions that strengthened the impact of the applied app techniques on PA performance. Reward (i.e., collecting points) did not influence PA in one study [115], since users perceived receiving points as insufficiently meaningful. Hence, low performance expectancy renders app techniques ineffective. Results from two studies [123, 132] were mixed, as users followed-up on the intervention despite their concerns related to the reliability of the app. From a vote counting perspective, among these 14 qualitative studies, 12 versus two [123, 132] provided supportive evidence for a moderating effect of perceived usefulness. Combining this with the meta-synthesis results, evidence is strong that perceived usefulness plays a moderating role in the relationship between app design and PA.

#### Attitude Toward Apps

In total, 36.5% (*n* = 15/41) studies analyzed attitudes toward apps as another variable, studying attitudes regarding (1) playfulness, joyfulness, and practicability of the app and its techniques or (2) general positive attitudes toward it [17, 18, 55, 59, 75, 94, 98, 116, 117, 119, 121, 125, 130, 137, 139].

These studies, all qualitative, indicate the moderating role of attitude toward the app on the relationship between app techniques and PA. When users have positive attitudes toward apps or app techniques (vs. negative), the relationships between app techniques and PA seem stronger (weaker). For instance, effects of social sharing, competition, and feedback on PA were reported [75] as stronger, due to users’ positive attitudes toward these techniques and overall positive attitudes toward the app. Some even call the app “addictive” [121]. More than half the users in one study [116] performed PA and reported a positive attitude toward the app, since they evaluated it as suitable. Regarding vote counting, all 15 qualitative studies provide supportive evidence for the moderating role of attitude toward the app, supporting the results of meta-synthesis.

#### Perceived Barriers

In total, 9.8% (*n* = 4/41) of studies analyzed perceived barriers related to general mobile usage, such as faster battery depletion, the necessity of carrying the mobile phone, and difficulty making or receiving calls [116, 118, 123, 132].

The studies produced mixed results. Two qualitative studies indicate a moderating effect [116, 118]. When users reported perceiving lower barriers, the effects of app techniques on PA were stronger. In contrast, the qualitative results of [123, 132] could not support this notion. Users in one study [123] experienced difficulty making or receiving calls, and other users [132] had difficulty self-monitoring their performed PA. But these barriers did not prevent them from using the app; in other words, these issues did not weaken the effects of the app techniques on PA. The reason for these findings is that users in these two studies [123, 132] perceived the apps as very useful and aimed to continue using them despite the barriers. However, barriers for users in two other studies [116, 118] were core difficulties, including the necessity of carrying the mobile phones and problems with battery depletion, making it challenging to follow the interventions. From a vote counting perspective, while two qualitative studies [116, 118] provided supportive evidence, two other qualitative studies [123, 132] provided non-supportive evidence of a moderating role of perceived barriers. Hence, both meta-synthesis and vote counting produce mixed results for moderating effects of perceived barriers.

#### Motivation

In total, 22.0% (*n* = 9/41) of studies explicitly considered motivation [59, 75, 89, 117, 118, 121, 126, 131, 135], referring to intrinsic and extrinsic motivations. Specifically, motivation to perform PA was related to appearance, health, fitness, competence, challenge, and enjoyment. The studies indicate a mediating role for motivation. For instance, more than half the users in one study [117] claimed that the app motivated them toward a healthier life, which further led to performing more PA. For other users [59, 75, 118, 121], enjoyment, health, and fitness were important motivations leading them to foster PA. Overall, while studies considered different motivations and PA with different methodological approaches and designs (e.g., descriptive pre–post comparison or less systematic approaches, such as interview results), they generally indicated a mediating effect of motivation on PA. These qualitative findings were complemented by a quantitative result [89] that found effects of competition on PA were partially mediated by intrinsic motivation. From a vote counting perspective, eight qualitative studies [59, 75, 117, 118, 121, 126, 131, 135] and one quantitative study [89] provided supportive evidence.

#### Self-Efficacy

In total, 17.0% (*n* = 7/41) of studies considered self-efficacy. They looked at how self-efficacy changed (1) based on app usage in general and (2) because of specific app techniques, particularly feedback, reward, and competition [18, 55, 89, 94, 117, 130, 135]. Across the studies, we observed an indication of a mediating role of self-efficacy that appeared to act in two ways. For some users, app usage increased awareness (i.e., users reported that they realized their real ability to perform PA through using the apps) [117, 130]. For others, app usage made them confident of having control to perform PA. These users attempted to develop self-efficacy by trying to change old routines and developing new plans and sticking to them to perform (more) PA. The apps made them active in finding time to perform PA. For instance, one user [18] claimed, “‘Oh man, you are not anywhere near [your goal]. You better go take a walk.’ And so I would.” Another user [94] cancelled her daily newspaper delivery to find time to walk more. These qualitative interview studies and interview results [55] support the idea that perceived self-efficacy can increase through app usage, and increased self-efficacy can result in higher PA. In fact, feedback and reward represent some sort of personal success to users that raises their belief in possessing the capability to master PA. However, one non-supportive study [89] also reported a non-significant mediation effect of self-efficacy of competition in PA, perhaps due to how the competition was designed in the study. Regarding vote counting, six qualitative studies [18, 55, 94, 117, 130, 135] provided supportive evidence, while one quantitative study [89] failed to provide evidence. Hence, meta-synthesis and vote counting together mostly indicate a mediating role of self-efficacy. These findings should be considered tentative, given the lack of quantitative support and the findings not covering all app techniques.

### Physical Activity

PA was considered at all three levels—light, moderate, and vigorous—with 12.1% (*n* = 5) considering PA at light levels, 80.4% (*n* = 33) at moderate levels, and 44.0% (*n* = 18) at vigorous levels. Another 4.9% of studies (*n* = 2) did not specify the PA level. Most studies focused on more than one level, resulting in the overall combined reported numbers exceeding 100%.

### Demographic Variables

Interestingly, none of the studies analyzed sex, age, and educational level as moderating variables on the effects of app techniques on PA. Although not considered by our conceptual framework, six studies analyzed the direct effects of these variables on PA. But neither sex, age, nor educational level exhibited explanatory power [17, 19, 115, 122, 123, 129], except in one study [129], where females increased steps/day significantly more than males.

### Summary of Results

**Fig. 3:**
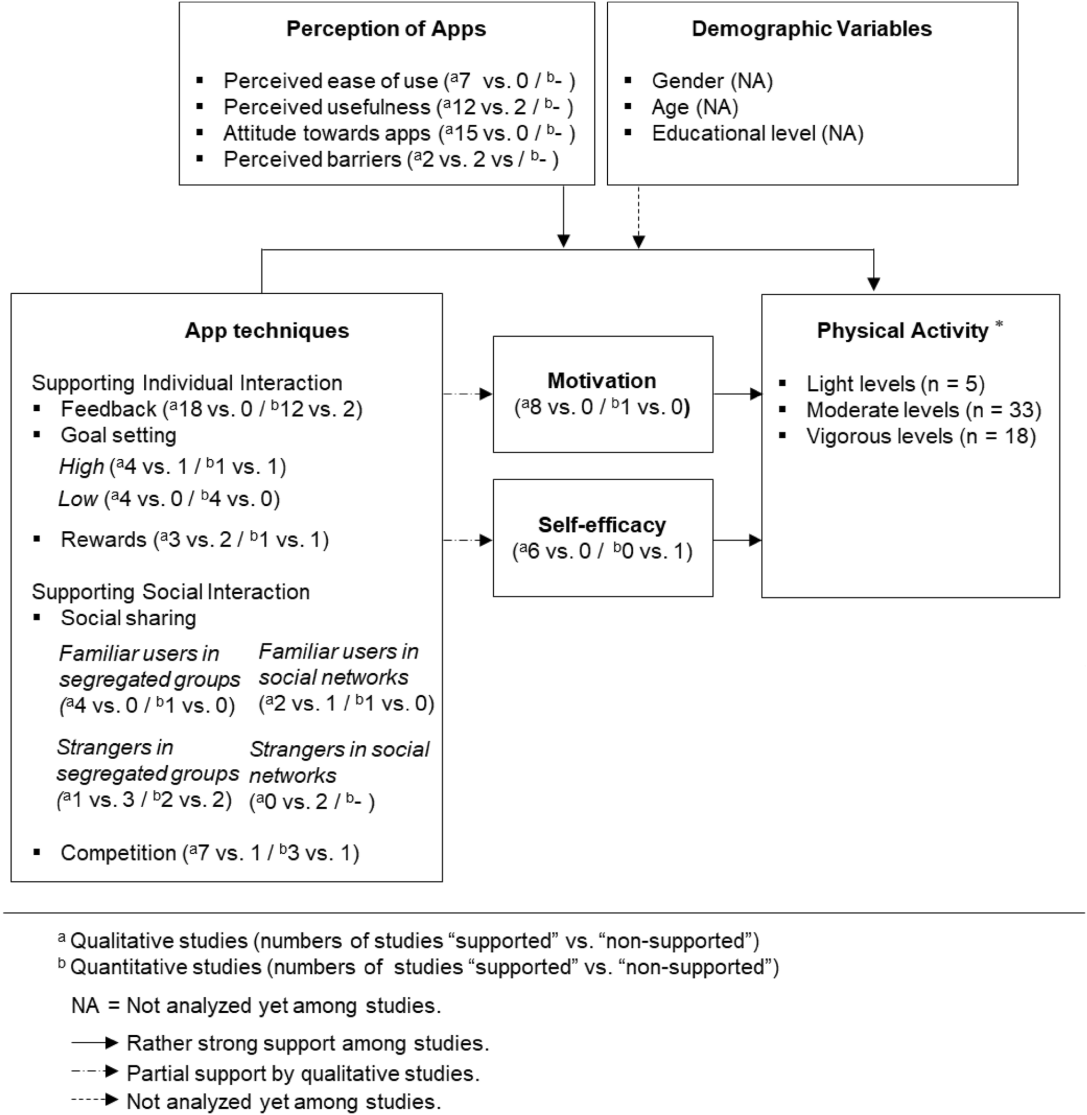
Summary of the results. Asterisk: most studies focused on more than one level, resulting in the overall combined reported numbers exceeding 41. Two studies did not specify the level of physical activity

Figure 3 summarizes the results of our study. For each construct, we provide the vote counting results as a ratio of supportive versus non-supportive evidence in the qualitative and quantitative studies. The results reveal that the app techniques feedback, goal setting and its sub-forms (with a slight tendency favoring less over more challenging goals), competition, and social sharing with familiar users in segregated and social network groups foster PA. Rewards and social sharing with strangers in segregated and social network groups produced mixed results. Results also indicated that the effects of app techniques on PA were stronger with higher perceived ease of use, higher perceived usefulness, and a more positive attitude toward the app. Motivation and self-efficacy also foster PA. There is some indication that motivation mediates the effects of feedback, goal setting (both high and low levels), reward, social sharing (only when sharing with familiar users in both segregated and social network groups), and competition on PA. Self-efficacy seems to be a mediator for the effects of feedback and may be for reward on PA. A possible moderating role of demographic variables was not analyzed in the studies. We would also like to point out that the research field is characterized by unelaborated theoretical development and in terms of methodology by many qualitative and few quantitative studies.

## Discussion

We attempted to provide a theoretically guided review of the effectiveness of specific app techniques to foster users’ PA behavior and to shed more light on why some apps can foster PA while others cannot. The present analysis was also deemed to provide an updated review of the literature. With a total of 41 studies, it covers a more comprehensive study base than prior reviews, which is desirable when aggregating research findings [141]. Results of the present summary offer four main contributions.

First, by applying meta-synthesis and vote counting approaches, we provide an integrative account of supporting and non-supporting evidence of app techniques’ ability to foster PA for five techniques and their sub-forms [16, 19, 56, 59, 89, 115, 126], as far as they have been examined in the literature to date. The app techniques include feedback, goal setting (sub-forms: app vs. user vs. mixed-set goals; high vs. low goal challenge levels), reward, social sharing (sub-forms: with familiar users in either segregated or social network groups or with strangers in either segregated or social network groups), and competition. The results are summarized in Table 2.

**Table 2:** Summary of key results and conclusions for the effectiveness of the app techniques

Results (supported vs. non-supported)		Conclusion
Feedback		
Qual. Quant.	18 vs. 0 12 vs. 2	Presence of feedback was the app technique most often studied; in all but one study, it was supported to foster PA. Hence, feedback is an effective and robust app technique in promoting PA
Goal setting^a^		
High		
Qual. Quant.	4 vs. 1 1 vs. 1	Presence of high goals was fairly often studied and supported to foster PA in all but two studies. Hence, based on these results, goal setting with challenging goals seems for the most part an effective app technique in promoting PA
Low		
Qual. Quant.	4 vs. 0 4 vs. 0	Presence of low goals was often studied and supported to foster PA in all studies. Hence, based on these results, goal setting with less challenging goals is an effective app technique in promoting PA, which is probably more effective than challenging goals
Reward		
Qual. Quant.	3 vs. 2 1 vs. 1	Presence of reward was fairly often studied, and results were mixed. Based on this, we cannot yet draw definite conclusions about its effectiveness in fostering PA. Additional research that explains the mixed findings is needed
Social sharing		
Familiar users in segregated groups		
Qual. Quant.	4 vs. 0 1 vs. 0	Presence of social sharing with familiar users in segregated groups was occasionally studied and, in all studies, supported in fostering PA. Hence, this social sharing type appears promising as an effective app technique for promoting PA
Familiar users in social networks		
Qual. Quant.	2 vs. 1 1 vs. 0	Presence of social sharing with familiar users in social networks was one of the least studied techniques, and in three studies versus one, was shown to foster PA. Thus, pending future research, this social-sharing type might also be an effective app technique for promoting PA
Strangers in segregated groups		
Qual. Quant.	1 vs. 3 2 vs. 2	Presence of social sharing with strangers in segregated groups was often studied. In three studies, it was supported as fostering PA; in five, not. Hence, results indicate that this sharing type seems rather ineffective but may work under special conditions that need to be identified in additional research
Strangers in social network		
Qual. Quant.	0 vs. 2 –	Presence of social sharing with strangers in social networks was one of the least often studied app techniques, and in two studies, it was not found to be a driver of PA, with particularly strong negative reactions of users. Hence, this social sharing type is, pending research, probably not effective in promoting PA
Competition		
Qual. Quant.	7 vs. 1 3 vs. 1	Presence of competition was often studied, and all but two studies supported it as a driver of PA. Hence, results render competition for the most part an effective app technique in promoting PA

*PA* physical activity, *Qual.* qualitative, *Quant.* quantitative

^a^Presence of app-set goals, user-set goals, and a mix of app-set and user-set goals is merely a design element

Based on the ratio of supportive versus non-supportive evidence in the qualitative and quantitative studies, we propose the following descending rank order for the effectiveness of app techniques to foster PA (tentative in nature, because of the overall still small body of literature, rendering definite conclusions difficult): (1) feedback, (2) goal setting and its sub-forms, with a slight tendency favoring less over more challenging goals, (3) competition and social sharing with familiar users in segregated and social network groups, and (4) rewards and social sharing with strangers in segregated and social network groups. In particular, rewards, but also social sharing with strangers in segregated and social network groups, provided mixed results. These findings provide important advances in current knowledge. While prior reviews have been inconclusive about the effectiveness of apps in fostering PA, present results suggest that mixed findings are at least partly explained by different techniques and sub-forms implemented in an app. As such, our results indicate that an app that provides the right techniques (i.e., feedback, goal setting, social sharing with familiar users either in segregated or social network groups, and competition) is more likely to be effective in promoting PA.

Vote counting results showed mixed evidence for social sharing with strangers in segregated and social network groups and for rewards. Meta-synthesis revealed that users in general felt social sharing with strangers was awkward, unnecessary, and uncomfortable, making social sharing with familiar users more promising. Particularly strong negative reactions were observed for social sharing with strangers in social networks, and hence, it seemed ineffective in fostering PA. Regarding rewards, the significant absence of supportive results was surprising but may be explained by rewards being subject to wear-out effects and users indicating they considered rewards meaningless [115] and gimmicky or felt disappointment in not receiving them [98]. One study [128] examined rewards quantitatively, reporting positive significant effects attributed to how users mentioned rewards as increasing their interactions with the app and perceived self-efficacy. Additional research into the rewards and the types used is certainly required to gain a deeper understanding of their role in fostering PA via apps.

Second, considering a lack of theoretical integration in prior reviews, we contribute a theoretical framework for analyzing app technique effects on PA. The proposed framework is guided by BCT taxonomy [25], in which the techniques (feedback, goal setting, rewards, social sharing, and competition) foster PA, partially mediated by motivation and self-efficacy and moderated by perceptions of app techniques. We also considered the moderating roles of demographic variables. To assess the relationships among the variables in our proposed conceptual framework, we leveraged vote counting and meta-synthesis. The results of both indicate that the suggested framework was useful for our systematic review.

Feedback and goal setting yielded positive effects on PA. These findings are in line with control theory, which proposes that people who are goal driven respond to feedback about divergence between their performance and their goal [37], at least as long as the goals are still perceived as doable. Likewise, social sharing with familiar users and competition showed mostly positive effects on motivation and PA. This result supports the theoretical argument derived from social cognitive theory that people learn to accomplish a specific behavior by socially interacting and observing others [43, 97, 142].

Motivation is also an important determinant of PA behavior, explained by self-determination theory, which suggests that intrinsic and extrinsic forces drive people to perform PA [143]. In line with social cognitive theory, self-efficacy can also contribute to performing more PA. The review revealed that self-efficacy seems to be a mediator for feedback and may be for reward techniques, whereas evidence for the other techniques is insufficient. Finally, we observed that high perceived ease of use, high perceived usefulness, and positive attitude toward apps may strengthen the effects of app techniques on PA. These results are in line with technology-acceptance models. We also found some indication that lower perceived barriers may strengthen the effects of app techniques on PA, but studies rarely examined this effect.

The proposed framework proved feasible for integrating prior research findings. It also allowed an integration of a set of theories relating to specific model elements. Therefore, it may serve as theoretical guidance for pending research, which appears to be needed. Aligning theory and study objectives is crucial to fully exploit the potential of app-facilitated PA. Theoretical alignment can provide guidance on how app techniques should be designed to change behavior and build stronger tests for pending discoveries.

Our third contribution is to provide a comprehensive, up-to-date tabular description of the 41 individual studies (ESM 1). This overview will help future researchers get a structured overview of the research field, and it supports comparisons of different studies.

As outlined in the results section (Table 1), users’ demographic characteristic distributions across studies favored male respondents and high-income nations. Regarding methodological approaches, most findings were qualitative in nature, reflected in the sample size distribution, where larger samples of ≥ 70 were clearly underrepresented (29.4 %). This information provides contextual details for the generalizability of findings. Importantly, while the obtained results may hold true for the overrepresented groups, the findings should be transferred only with caution to other target groups (females, low- and middle-income nations).

Finally, in our fourth contribution, we attempt to identify avenues for future research that are, at the same time, related to limitations of the present review. First, the characterization of studies reveals a need for studies conducted in low- and middle-income nations, which we—in line with Bort-Roig et al. [15]—encourage. There is a need to focus more on these nations, since rates of physical and mental health problems are rising in these countries [144]. Given the prevalence of smartphones worldwide, apps may provide opportunities to reach traditionally underprivileged groups. These could include individuals with poor healthcare and delivery related to their demographic, geographic, or economic background. Elderly individuals—given that their smartphone usage is also rising—with low socioeconomic status and individuals with disabilities are some of these groups [7]. One study [145] also considered immigrants to Western countries as target groups for app interventions, given their insufficient PA. Further, given the smaller share of females in the reviewed studies (42.9 %), a stronger concentration on female samples is needed. Females are typically more concerned with their health than males but also often have higher anxiety levels concerning technology [100] and seem less accepting of new technological devices in general [99]. Finally, more quantitative studies are strongly advised. Later, and in a second step, quantitative investigations would provide the opportunity to meta-analytically integrate the research findings, which would allow for a better calibration of the strengths of effects [146].

A second research avenue revolves around the app techniques; future research may analyze specific elements of current techniques or uncover new ones. Surprisingly, results of our current review revealed that rewards in the apps mostly failed to foster PA. Additional research is essential to discover in which ways and how rewards may foster PA, since many theories identify positive reinforcement as an important motivator for future behavior. It is also essential to discover means of overcoming users’ feelings of disappointment upon not receiving rewards. Future studies may also consider focusing on effects of graphical designs of apps on users’ engagement with them and thus on performing PA. Researchers could analyze whether different triggers in the form of textual, audio, or visual cues can affect PA differently, or they could focus on the appropriate time or schedule of rewards [147].

Third, future research may focus on the processes determining how techniques translate into PA. Studies may explore whether apps trigger internal or external motivation and which of those two pathways to PA is more salient or whether other mediators are conceivable. Studies may also focus on methods of increasing self-efficacy. Users need to learn they have the capability of performing PA. One method could be to make performing PA less difficult. To do so, studies may increase self-efficacy by trying to target low or moderate PA, setting light goals, and even engaging users with friends or family. The current field of research may also benefit from studies following an even more interdisciplinary approach. Future studies may draw on findings allowing insights into mechanisms or techniques to change behavior, from fields as diverse as sociology, politics, economics, marketing, and media research, which could be conceptually transferred to the present scope of investigation to derive additional insights about how to promote PA.

Another aspect that became apparent in our review was the need for researchers to develop more theoretical frameworks in their studies; theoretical development was relatively rare among studies. In our study, we applied three main theories in this field: control, social cognitive, and self-determination theory. We also investigated some variables related to the technology-acceptance model. However, none of the studies comprehensively applied a technology-acceptance model. We suggest that future research make use of one of the more current models that have been developed in the field, such as the unified theory of acceptance and use of technology (UTAUT 1 or UTAUT 2) [76].

Another theory that may inform an understanding of effects of apps and app techniques on PA is the regulatory focus theory [151]. This theory distinguishes between two distinct regulatory orientations of individuals: promotion focus and prevention focus. People with a promotion focus are sensitive to the absence and presence of positive outcomes; they are concerned with growth, accomplishments, and aspirations. In contrast, those with a prevention focus are sensitive to the absence and presence of negative outcomes; they are concerned with safety, responsibilities, and obligations. According to this theory, any goal may be pursued with either a promotion or a prevention focus, including performing PA via apps. For instance, should the app’s feedback on PA depict a promotion goal (e.g., an avatar getting healthier with increasing PA) or a prevention goal (e.g., the avatar is getting sicker and sicker with decreasing PA)?

Fourth, mid- or long-term effects of apps in promoting PA is another important issue for future studies to consider. Our review is limited in providing a conclusion on this matter, since only four studies mentioned such mid- or long-term effects [59, 119, 123, 137]. For instance, only 12 of 68 users [123] were willing to use the app after the intervention and only some in another study [119]. Other users [59, 137] also reported losing their initial excitement with the apps. Hence, studies on ensuring sustained app intervention effectiveness are strongly encouraged [7, 12]. One starting point might be investigating how app techniques can be better tailored to the user (personalization based on users’ characteristics) or how two-way communications and regular interactions with the app can be improved (e.g., an ongoing relationship with an app to set plans).

Fifth, while extant studies provide some evidence for the moderating roles of perceived ease of use, perceived usefulness, and attitude toward the app, the roles of perceived barriers and demographic characteristics are insufficiently examined. In particular, studies on the role of demographic variables are encouraged. Future research may explore additional variables amplifying or attenuating effects of app techniques on PA behavior. For instance, researchers may extend works on psychological determinants of PA, such as attitude toward PA, health consciousness, or perceived competence, that have been shown relevant for PA [148–150].

Finally, we tried to provide a comprehensive overview of the studies in the field by applying both meta-synthesis and vote counting. Meta-synthesis generates interpretative results aiming at understanding and aggregating key findings of studies, which is particularly challenging given that the studies often followed quite different approaches, views, and interpretations [108]. Hence, like other existing interpretative techniques aiming to synthesize and aggregate knowledge from heterogeneous studies, our meta-synthesis to some extent represents the authors’ point of view. The vote counting method aims to identify supportive, non-supportive, or conflicting results from different studies in a field on a highly aggregated level; it is often applied when the research field is still rather unstructured and when there is insufficient quantitative empirical evidence to perform a meta-analysis that aims at estimating effect sizes. Once the research field develops and more quantitative studies emerge, future studies may aim at estimating the strength of the effects.

The results of this review also offer some practical implications for healthcare providers and app developers in the area of eHealth and mHealth. Since our results indicate that certain app techniques seem to be more advantageous to stimulate PA, particularly feedback, goal setting (with more preference towards low levels), competition, and social sharing with familiar users in segregated and social network groups, app developers and healthcare providers should pay special attention to including these techniques when designing apps. Our results further indicate that higher perceived ease of use, higher perceived usefulness, and more positive attitudes toward apps can strengthen the impact of the app techniques on PA. Hence, healthcare providers and app developers need to consider not only which app techniques to implement, but also how to implement them in order to be most beneficial to the user. This can be done, for instance, via market research among potential users. Market research can assist healthcare providers to understand whether users perceive the apps as easy to use and useful to them to perform PA and to gain insights into their evaluation of the app. Finally, even though perceived barriers produced mixed results, from a theoretical point of view, we advise healthcare providers and app developers to also consider this aspect.

## Conclusion

Being a major risk factor for the physical and mental health of people, PA plays an important role in the well-being of individuals and societies. The present summary indicates that to nurture PA behavior via apps, the app techniques feedback, goal setting, competition, and social sharing with familiar users in segregated and social network groups seem to be techniques able to stimulate PA behavior, whereas social sharing with strangers in segregated and social network groups and reward were less effective. In terms of theoretical guidance, the BCT taxonomy appeared valuable for the present review and is therefore recommended as a theoretical framework for future studies.

Given the health-related implications of insufficient PA, apps may have an impressive potential to contribute to individual and social well-being. Hence, the strong interest of researchers and society in apps promoting PA is likely to grow in coming years. Having established the usefulness of apps in promoting PA, we believe that to fully exploit the potential of promoting PA via apps, the next research steps should (1) seek to provide further evidence about how app techniques’ design impacts PA; (2) process explanations about how app design translates into motivation and behavior and how effect size can estimate the quantifying of apps’ power to shape PA; and (3) provide insights into how situational contingencies favor or disfavor app-facilitated PA behavior. Additional research is needed to explore mid- and long-term effects of app use on PA, and research should also cover less developed countries. Finally, a better theoretical development of the field is recommended.

## Electronic supplementary material

Below is the link to the electronic supplementary material.

Supplementary material 1 (DOCX 113 kb)
