# Supplementary material for: Your Personal Motivator is with You: A Systematic Review of Mobile Phone Applications Aiming at Increasing Physical Activity
Source: Sports Med. 2019 May 29;49(9):1425–47. doi: 10.1007/s40279-019-01128-3 (PMC6684571; doi:10.1007/s40279-019-01128-3)
Supplement: Supplementary file 1 — Supplementary material 1 (DOCX 113 kb) [file 40279_2019_1128_MOESM1_ESM.docx]

Electronic Supplementary Material 1

Electronic Supplementary Material 1 Continued

continued

Electronic Supplementary Material 1 Continued

Electronic Supplementary Material 1 Continued

Electronic Supplementary Material 1 Continued

Electronic Supplementary Material 1 Continued

Electronic Supplementary Material 1 Continued

Electronic Supplementary Material 1 Continued

Electronic Supplementary Material 1 Continued
